# Design and synthesis of analogues of RA-VII—an antitumor bicyclic hexapeptide from Rubiae radix

**DOI:** 10.1007/s11418-021-01542-w

**Published:** 2021-07-10

**Authors:** Yukio Hitotsuyanagi

**Affiliations:** Department of Natural Products and Medicinal Chemistry, School of Pharmacy, Tokyo University of Pharmacy and Life Sciences, 1432-1 Horinouchi, Hachioji, Tokyo 192-0392 Japan

**Keywords:** Analogue synthesis, Cytotoxic activity, Cyclic peptide, Cycloisodityrosine, *Rubia* species, Rubiaceae

## Abstract

The 14-membered cycloisodityrosine is the core structure of RA-series antitumor bicyclic peptides obtained from *Rubia* plants (Rubiaceae). In this study, an efficient method for the synthesis of cycloisodityrosines from commercially available l-tyrosine derivatives was developed. Using synthetic cycloisodityrosines and cycloisodityrosines with modified structures, several RA-VII analogues were designed and synthesized to explore structure–activity relationships of the cycloisodityrosine moiety of the RA-series peptides, and newly isolated natural peptides were synthesized to establish their structures.

**Graphic abstract:**

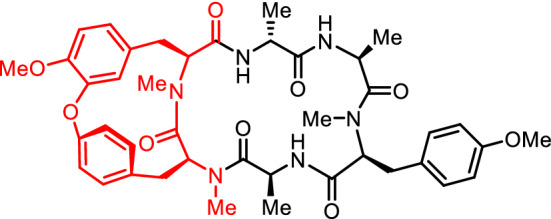

## Introduction

**Fig. 1:**
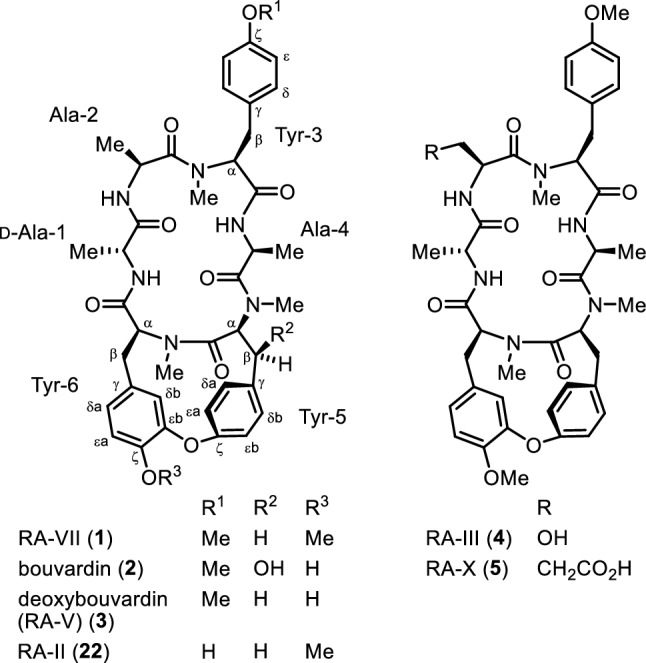
Structures of RA-series peptides and bouvardins

RA-VII (**1**) is a bicyclic hexapeptide isolated from the roots of rubiaceous plants *Rubia cordifolia* L. and *Rubia argyi* (H. Lév. & Vaniot) H. Hara ex Lauener & D.K. Ferguson (syn. *Rubia akane* Nakai), which are known as Rubiae radix [1, 2]. Two structurally related peptides, bouvardin (NSC 259968) (**2**) and deoxybouvardin (RA-V) (**3**), were isolated from *Bouvardia ternifolia* (Cav.) Schltdl., a plant of the same family (Fig. 1) [3]. These peptides exhibit potent antitumor activity; peptide **1** underwent phase I clinical trials as an anticancer drug in Japan from the late 80s to the early 90s [4, 5]. The antitumor activity of these peptides is believed to be due to the inhibition of protein synthesis through interaction with eukaryotic ribosomes [6–8]. Peptide **1** was also shown to cause conformational changes in F-actin, which stabilize actin filaments and induce G2 arrest [9], and peptide **3** was found to inhibit angiogenesis by downregulating ERK1/2 phosphorylation in HUVEC and HMEC-1 endothelial cells [10].

These peptides have a structurally unique cycloisodityrosine unit in which the two phenyl rings of tyrosyl-tyrosine are connected by an ether linkage to form a 14-membered cyclophane macrocycle, and a few compounds of this type are known among natural products. In these peptides, the ether linkage is formed between the carbon atom at the ζ position of the N-terminal tyrosine and the carbon atom at the ε position of the C-terminal tyrosine of the cycloisodityrosine unit. We have synthesized various RA-VII analogues by the chemical transformation of natural RA-series peptides, including RA-VII (**1**), deoxybouvardin (**3**), RA-III (**4**), and the methyl ester of RA-X (**5**), or by semi-synthesis using the cycloisodityrosine obtained by the degradation of RA-VII (**1**), and those analogues have been described in previous reviews [2, 11]. This paper provides a summary of our subsequent research studies of RA-VII analogue synthesis and natural RA-series peptide synthesis utilizing the totally synthetic cycloisodityrosines prepared by our method.

## Synthesis of cycloisodityrosine

The 14-membered cycloisodityrosine is difficult to prepare due to its strained cyclophane structure and the presence of an easily epimerizable chiral center on the C-terminal tyrosine [12, 13]. We previously developed a method to obtain the 14-membered cycloisodityrosine by degradation of natural RA-VII (**1**) [14, 15]. This transformation proceeded efficiently, and several RA-VII analogues in which d-Ala-1, Ala-2, or Ala-4 of **1** was replaced by another amino acid residue were synthesized using this degradation product [15–18]. The cycloisodityrosine derived from the natural RA-series peptide inevitably has an *N*-methyl group at both tyrosine moieties, and conditions that can be used for the chemical modification of this cycloisodityrosine is limited due to its rather stereochemically labile nature, which makes the synthesis of analogues of **1** with a modified cycloisodityrosine structure difficult. To synthesize analogues with a structurally modified cycloisodityrosine, a total synthesis of the 14-membered cycloisodityrosine was sought.

**Fig. 2:**
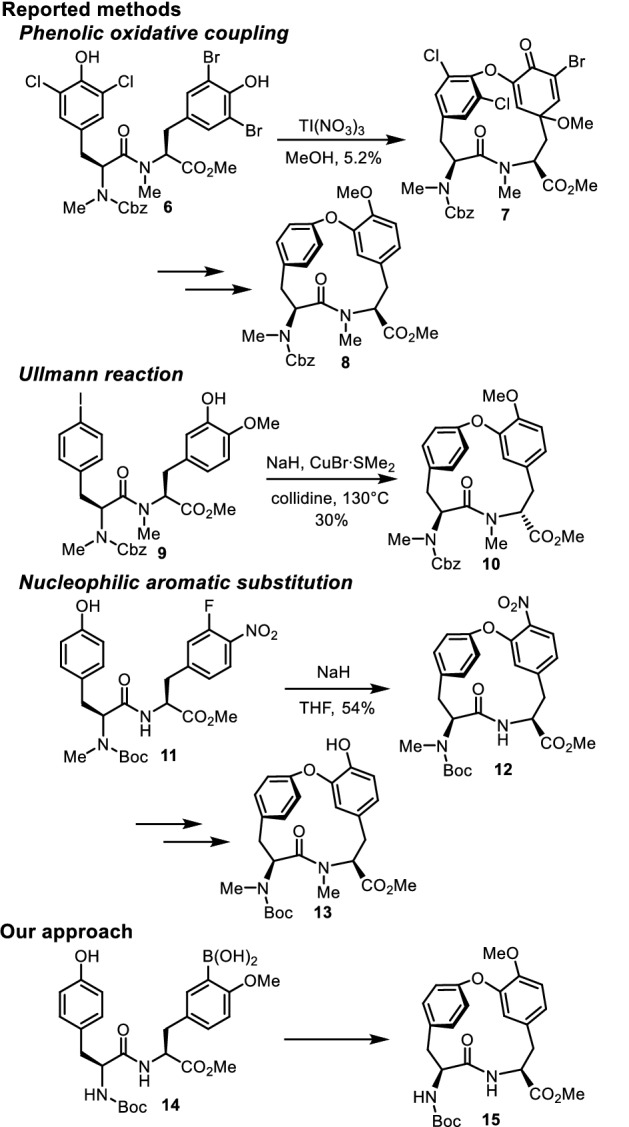
Methods for the synthesis of cycloisodityrosines

In the synthesis of cycloisodityrosines, the formation of the diphenyl ether linkage is a crucial step. Harsh conditions often used for the diphenyl ether formation have resulted in the epimerization of the C-terminal tyrosine. Several methods are available for the synthesis of 14-membered cycloisodityrosines (Fig. 2). The first total synthesis of the cycloisodityrosine of RA-VII was accomplished by Inoue and co-workers, who used an intramolecular phenolic oxidative coupling reaction for the diphenyl ether synthesis. They treated protected dipeptide with N-terminal 3,5-dichloro-*N*-methyl-l-tyrosine and C-terminal 3,5-dibromo-*N*-methyl-l-tyrosine **6** with thallium(III) nitrate to obtain cyclic intermediate **7** albeit in low yield, and then reduced **7** to generate cycloisodityrosine **8** [19]. The second approach to the synthesis of cycloisodityrosine is based on an intramolecular Ullmann reaction. Boger and Yohannes synthesized cycloisodityrosine through the Ullmann reaction of protected dipeptide with N-terminal 4-iodo-*N*-methyl-l-phenylalanine and C-terminal 3-hydroxy-*N,O*-dimethyl-l-tyrosine **9** [20]. Although this reaction proceeded in moderate yield, obtained cycloisodityrosine **10** was proven later to possess an undesirable unnatural configuration [12]. Epimerization at the α-carbon of the C-terminal tyrosine occurred on the cyclized product under the reaction conditions employed. The third approach consists of an intramolecular nucleophilic aromatic substitution reaction for the formation of a diphenyl ether bond, which was originally devised by Zhu et al. [21]. Treatment of protected dipeptide with N-terminal *N*-methyl-l-tyrosine and C-terminal 3-fluoro-4-nitro-l-phenylalanine **11** with sodium hydride afforded cyclized intermediate **12**, and subsequent conversion of its nitro group into a hydroxy group gave cycloisodityrosine **13** [22]. Although the pivotal diphenyl ether bond formation step of this sequence proceeded effectively, the preparation of the dipeptide substrate for this reaction required the asymmetric synthesis of a chiral unnatural amino acid.

**Fig. 3:**
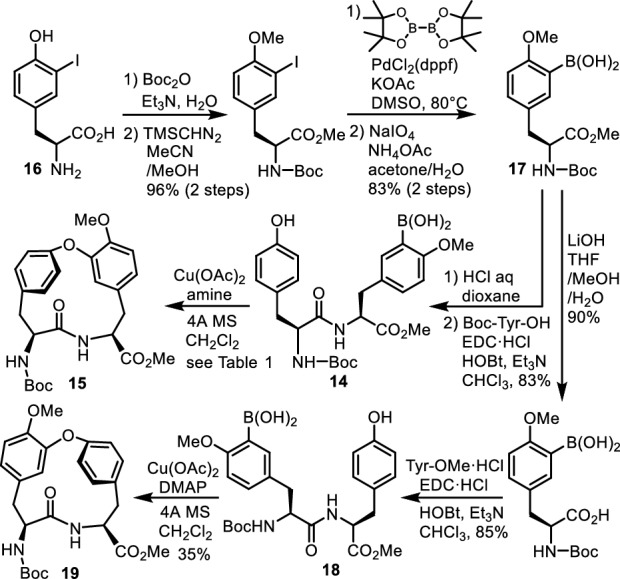
Synthesis of cycloisodityrosines **15** and **19**

We developed a short synthetic route to a cycloisodityrosine from commercially available l-tyrosine derivatives. Chan et al. [23] reported a coupling reaction between phenol and aryl boronic acid under very mild conditions to form diphenyl ether compounds, and Evans et al. [24] reported an arylation of the tyrosine hydroxy group using this method. We applied this phenol/aryl boronic acid coupling in an intramolecular fashion to construct the 14-membered cycloisodityrosine ring, as illustrated by the conversion of dipeptide **14** into **15**. When dipeptide **14**, which is obtained by the coupling reaction of Boc-l-tyrosine and 3-boryl-4-methoxy-l-tyrosine methyl ester (Boc-deprotected **17**) prepared from commercially available 3-iodo-l-tyrosine (**16**), was treated with 1 equiv of copper(II) acetate and 5 equiv of pyridine in dichloromethane at the concentration of 0.025 M, cycloisodityrosine **15** was produced in 29% yield. Optimization of the reaction conditions revealed that this reaction best proceeds with 4-(dimethylamino)pyridine (DMAP) instead of pyridine, at the substrate concentration of 0.013 M in an acceptable yield of 56% (Fig. 3; Table 1). This method was also effective for the synthesis of isomeric cycloisodityrosine **19**, which was produced in 35% yield from dipeptide **18**, by employing the same conditions used for the formation of **15** [25]. This isomeric cycloisodityrosine structure was seen in the structure of RP-66453, a secondary metabolite from *Streptomyces* sp. [26], and also in the structure of allo-RA-V (**20**) described later.

**Table 1:** Cyclization of dipeptide **14**

Entry	Amine	Conc (M)	Yield (%)
1	Pyridine	0.025	29
2	Triethylamine	0.025	5
3	*N,N*-Diisopropylethylamine	0.025	4
4	3,5-Dichloropyridine	0.025	6
5	2,2′-Dipyridyl	0.025	4
6	1,10-Phenanthroline	0.025	12
7	4-Picoline	0.025	36
8	4-(Dimethylamino)pyridine	0.025	45
9	4-(Dimethylamino)pyridine	0.013	56
10	4-(Dimethylamino)pyridine	0.0063	55

1 equiv of Cu(OAc)_2_, 5 equiv of amine, and powdered 4 Å molecular sieves in CH_2_Cl_2_ for 48 h at rt

## Synthesis of RA-VII analogues

Using the cycloisodityrosines synthesized by this method, three RA-VII analogues with modified tyrosine side chains were designed and synthesized to explore the effects of the aromatic ring of the tyrosine side chains on the cytotoxic activity.

### Bis(cycloisodityrosine) analogue

**Fig. 4:**
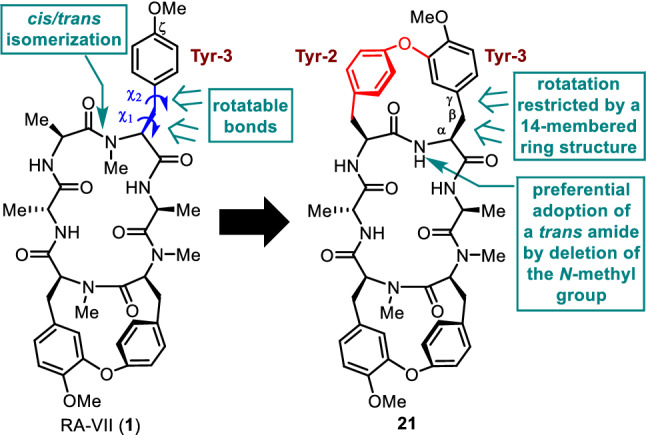
Structural design of analogue **21** in which the side-chain orientation of Tyr-3 residue was restricted by an additional cycloisodityrosine structure

**Fig. 5:**
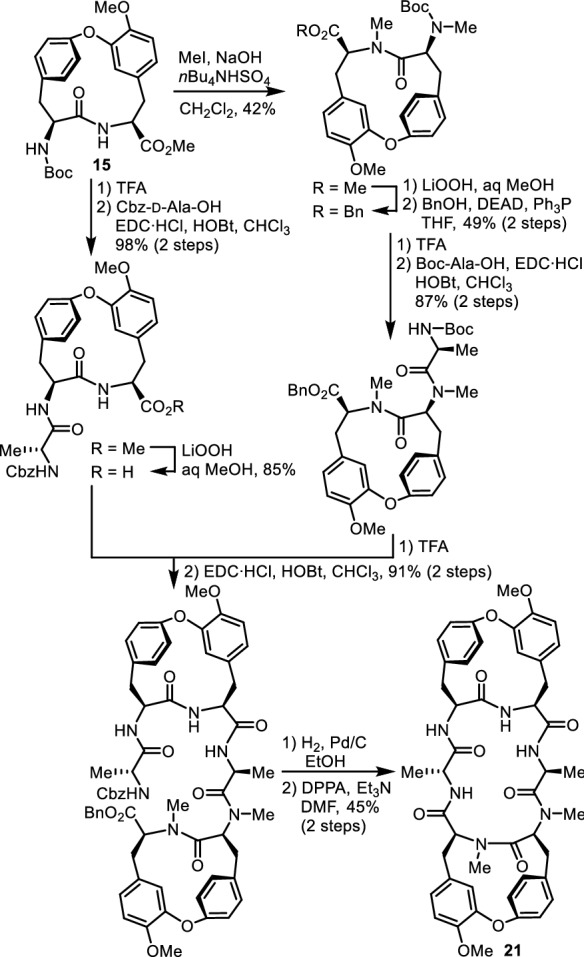
Synthesis of bis(cycloisodityrosine) analogue **21**

**Fig. 6:**
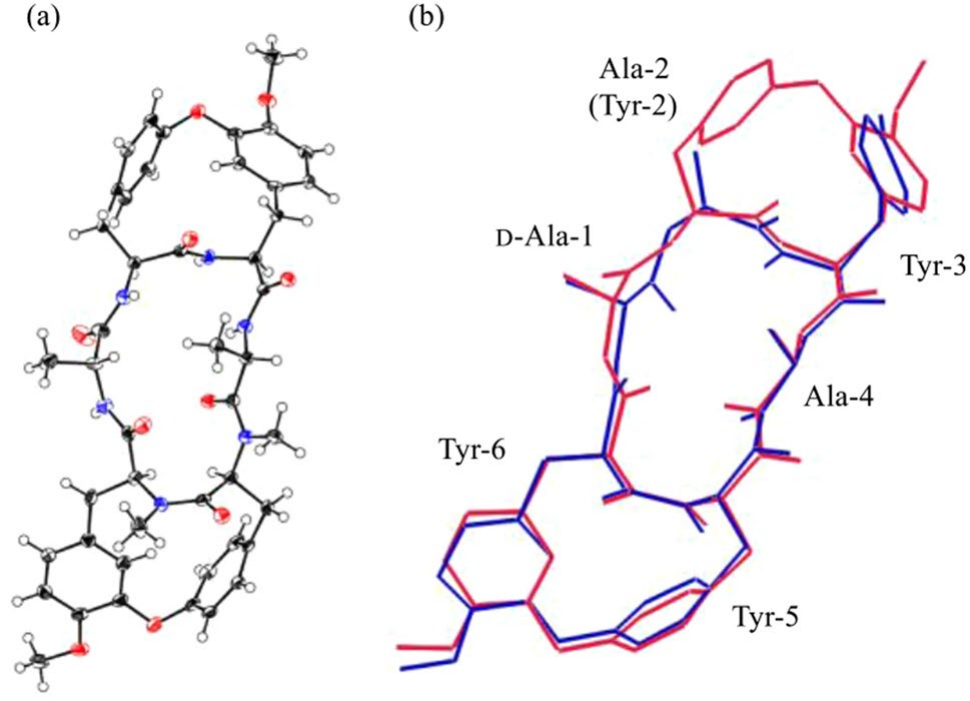
**a** Crystal structure of analogue **21** and **b** overlay of the crystal structures of RA-II (**22**) (blue) and analogue **21** (red)

Using the cycloisodityrosines prepared by this method, we first synthesized the bis(cycloisodityrosine) analogue of RA-VII (**1**). Of the three tyrosines at residues 3, 5, and 6 in peptide **1**, Tyr-5 and Tyr-6 constitute the cycloisodityrosine unit by forming a linkage between the ζ-oxygen atom of Tyr-5 and the ε-carbon atom of Tyr-6. Due to the planar amide bond and the 1,3-disubstituted and 1,4-disubstituted phenyl rings included in the 14-membered ring of the cycloisodityrosine unit, the rotation of the side chains of those residues is restricted. The remaining side chain at Tyr-3 rotates about the C_α_–C_β_ (*χ*_1_) and C_β_–C_γ_ (*χ*_2_) bonds. As the substituent at the ζ position of Tyr-3 is known to be greatly related to the activity [27], the *χ*_1_ and *χ*_2_ angles of Tyr-3, defining the spatial orientation of the Tyr-3 phenyl ring, appear to play a critical role in the cytotoxicity. To obtain information on the effect of the side-chain conformation of Tyr-3 on the activity, we designed an analogue having a restricted Tyr-3 side-chain rotation, which was realized by replacing the Ala-2 and Tyr-3 residues of **1** by a cycloisodityrosine unit, yielding bis(cycloisodityrosine) analogue **21** (Fig. 4). In the synthesis of analogue **21**, two tripeptide segments corresponding to d-Ala-1–Tyr-2–Tyr-3 and Ala-4–Tyr-5–Tyr-6 were each prepared from cycloisodityrosine **15**. The coupling reaction of these two segments and the subsequent macrocyclization of the resultant linear hexapeptide gave analogue **21** (Fig. 5). The similarity in the three-dimensional structural features of RA-VII (**1**) and **21** was highlighted by superimposing the crystal structure of **21** over that of RA-II (**22**), whose conformational property is known to be identical to that of RA-VII (**1**) (Fig. 6). The spatial positions of the phenyl rings of the three tyrosines and the peptide backbone conformation at residues 2–6 of peptides **21** and **22** are almost superimposable, indicating that analogue **21** may effectively mimic one of the lowest energy conformations in RA-VII including the side chain of Tyr-3.

Analogue **21** was 5000-fold less cytotoxic than RA-VII towards P-388 leukaemia cells. This result apparently does not agree with our hypothesis that the side-chain conformation at Tyr-3 of peptide **1**, as shown in the crystal structure of **22**, is a major factor that determines the cytotoxic activity of the compounds of this series. The bulky phenoxy tether connecting the β-carbon atom of Ala-2 and the ε-carbon atom of Tyr-3 in analogue **21**, however, may be hampering its necessary close access to the relevant binding site, resulting in the low cytotoxicity. Synthesis of other analogues and their analyses may offer further information to solve this problem [28].

### Fluorinated analogues

**Fig. 7:**
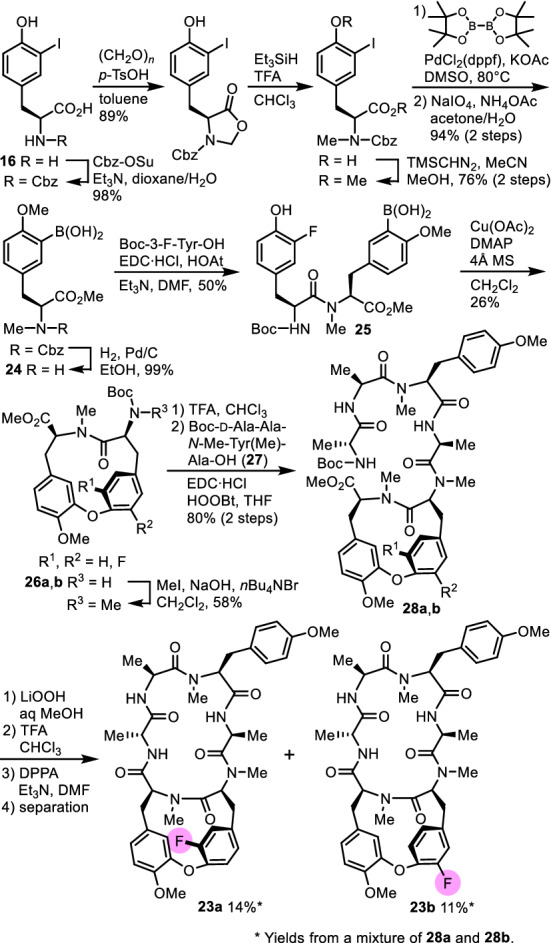
Synthesis of fluorinated analogues **23a** and **23b**

Examination of the metabolites of RA-VII (**1**) in bile juice of rabbits to which **1** was administered intravascularly revealed that specific *N*-demethylation of Tyr-3, *O*-demethylation, and hydroxylation at the aromatic rings of Tyr-3 and Tyr-5 occurred. Among them, the metabolites whose εa or εb position of Tyr-5 was hydroxylated showed markedly reduced cytotoxic activity, and such hydroxylation of the aromatic ring is considered a bioinactivation process of peptide **1** [29]. Therefore, we designed and synthesized RA-VII analogues **23a** and **23b**, in which the εa- or the εb-hydrogen atom of Tyr-5 of RA-VII (**1**) was replaced by a fluorine atom to prevent metabolic hydroxylation at these positions (Fig. 7). Dipeptide substrate **25** for the cyclization was synthesized by a coupling reaction of Boc-3-fluoro-l-tyrosine and 3-borono-*N,O*-dimethyl-l-tyrosine methyl ester **24** obtained from **16**. Treatment of **25** with copper(II) acetate and DMAP gave an inseparable mixture of atropisomers of fluorocycloisodityrosine, **26a** and **26b**, which were then *N*-methylated, deprotected, and coupled with tetrapeptide **27** to give a mixture of linear hexapeptides **28a** and **28b**. Subsequent macrocyclization of the mixture gave a mixture of **23a** and **23b**, which could be separated by HPLC. Analogue **23a** displayed cytotoxicity that was as potent as that of RA-VII (**1**) towards human promyelocytic leukaemia HL-60 and human colonic carcinoma HCT-116 cell lines, whereas **23b** was 5.6-fold and 11-fold less cytotoxic, respectively, than **1**. The replacement of a hydrogen atom by a fluorine atom imposes only subtle steric changes, whereas the electron-withdrawing nature of fluorine atom reduces the π-electron density of the phenyl ring through inductive effects. Thus, the results also indicated that the π-electron density of the Tyr-5 phenyl ring is not related to the cytotoxic activity [30].

### Aza-cycloisodityrosine analogue

**Fig. 8:**
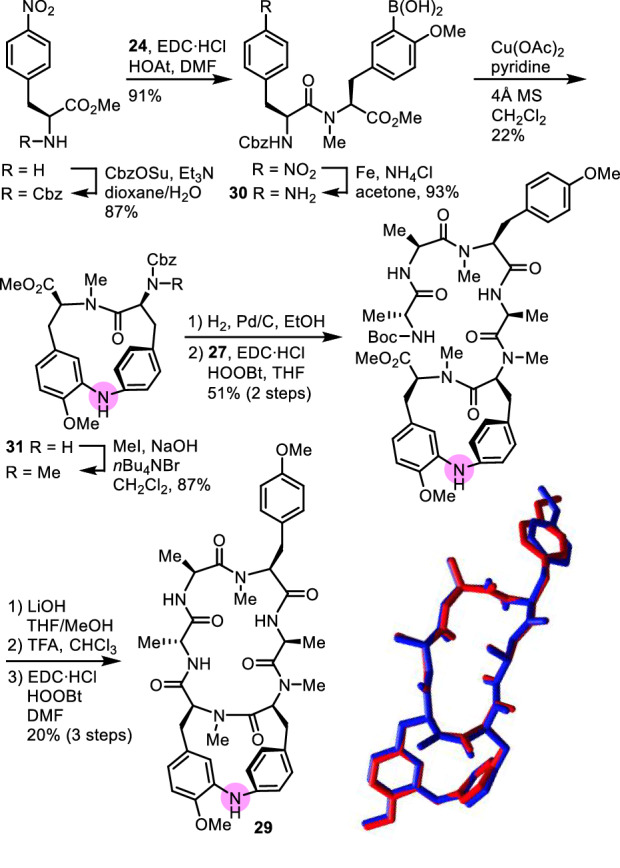
Synthesis of aza-cycloisodityrosine analogue **29** and superposition of the crystal structure of RA-VII (**1**, red) and the energy-minimized structure of analogue **29** (blue)

In the RA-series peptides, the cycloisodityrosine moiety is considered essential for the expression of the cytotoxic activity. This moiety not only affects the conformation of the 18-membered macrocycle of the peptides [31, 32], but also appears to participate in the expression of the activity [33, 34]. Thus, we sought to investigate the effect of the electron density of the phenyl rings of the cycloisodityrosine moiety on the cytotoxic activity. The electron density of an aromatic compound is often modulated by introducing an electron-donating or an electron-withdrawing group on the aromatic ring. A survey of a limited number of RA analogues having a substituent on the aromatic rings of the cycloisodityrosine moiety revealed that the introduction of a hydroxy group at the εa or εb position of Tyr-5, as mentioned in the previous section, or at the δa or εa position of Tyr-6 reduces the cytotoxic activity of RA-VII (**1**) [35]. Although such modification increases the electron density of the phenyl ring to which the hydroxy group is attached, it is not clear whether the reduction of the cytotoxic activity is due to changes in the electron density of the phenyl rings, the steric bulkiness of the introduced hydroxy group, and/or the polarity of the hydroxy group. It is also known that in RA-VII (**1**), the introduction of a hydroxy group at either the εa or εb position of the Tyr-5 residue affects the orientation of the cycloisodityrosine phenyl ring by forming a hydrogen bond between the hydroxy proton and the methyl ether oxygen of Tyr-6 [29]. Those conformational changes in the cycloisodityrosine moiety may be responsible for the reduced activity of those analogues. To obtain information on the effects of the electron density of the cycloisodityrosine phenyl rings in RA-VII on the cytotoxic activity, we designed RA-VII analogue **29**, in which the diphenyl ether oxygen of the cycloisodityrosine moiety was replaced by an amine nitrogen. We considered that this modification would increase the electron density of the phenyl rings of both Tyr-5 and Tyr-6 residues with minimal structural changes, as the steric demand between RA-VII (**1**) and analogue **29** is expected to be almost the same. Comparison of the energy-minimized structure of analogue **29** obtained by the Monte Carlo conformational search with the X-ray crystal structure of RA-VII (**1**) indicated that their 3D structures were very similar and almost superimposable (Fig. 8).

The aza-cycloisodityrosine unit of **29** was prepared by a copper(II) acetate-mediated cyclization reaction of protected dipeptide with N-terminal 4-amino-l-phenylalanine and C-terminal 3-borono-*N,O*-dimethyl-l-tyrosine **30**. Thus obtained aza-cycloisodityrosine **31** was then converted into analogue **29** using a similar synthetic pathway to that for the synthesis of analogues **23a,b**.

Analogue **29** was found to be 7.2-fold and 5.2-fold less cytotoxic than RA-VII (**1**) towards HL-60 cells and HCT-116 cells, respectively. These results indicated that as RA-VII (**1**) and its more electron-rich aza-analogue **29** possess almost the same 3D structure, enhancement of the electron density of the phenyl rings in Tyr-5 and Tyr-6 may reduce the cytotoxic activity of this series of peptides [36].

## Synthesis of natural compounds

The cycloisodityrosine in the natural RA-series peptides generally has an ether linkage between the carbon atom at the ζ position of the N-terminal tyrosine (Tyr-5) and the carbon atom at the ε position of the C-terminal tyrosine (Tyr-6), and those two tyrosines are both *N*-methylated. However, new peptides whose cycloisodityrosine has different structural features from those of known RA-series peptides were isolated from the roots of *Rubia cordifolia* L., and their structures were established by the synthesis of them.

### RA-XXV and RA-XXVI

**Fig. 9:**
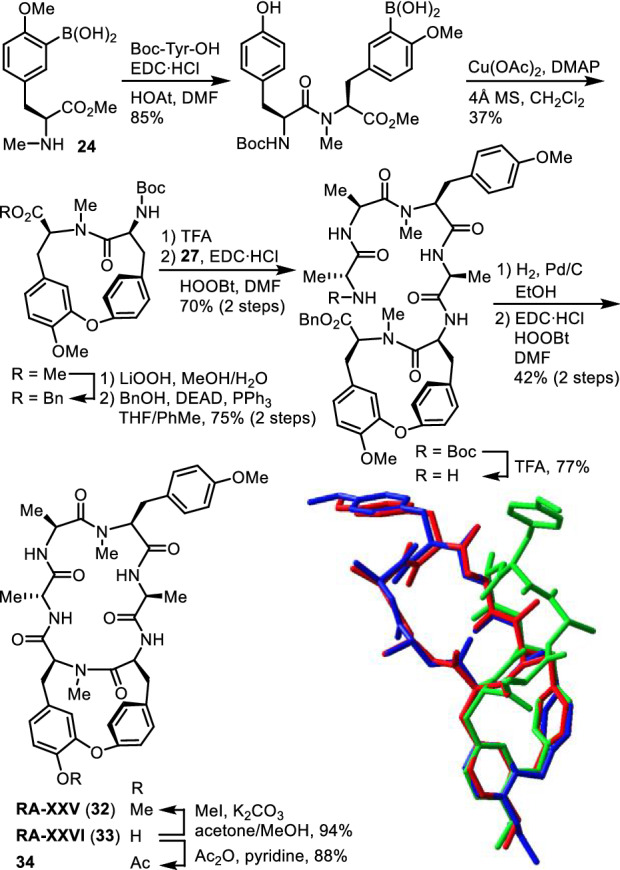
Synthesis of RA-XXV (**32**) and superposition of the crystal structures of RA-VII (**1**, red), RA-XXV (**32**, green), and RA-XXVI acetate (**34**, blue)

RA-XXV (**32**) and RA-XXVI (**33**) are des-*N*-methyl congeners of RA-VII (**1**) and deoxybouvardin (**3**) at Tyr-5, respectively [37]. Their amino acid compositions and sequences were determined by interpretation of MS as well as 1D and 2D NMR data, and their relative structures were elucidated by X-ray diffraction analysis of RA-XXV (**32**) and RA-XXVI acetate (**34**). The absolute stereochemistry of RA-XXV (**32**) was established by the total synthesis of it, and that of RA-XXVI (**33**), by the chemical correlation with **32**. Peptide **32** was synthesized by a route analogous to the synthesis of analogues **23a,b** and **29** (Fig. 9). The cytotoxic activities of RA-XXV (**32**) and RA-XXVI (**33**) on HL-60 and HCT-116 cell lines were significant but weaker than those of the corresponding *N*-methyl congeners. RA-XXV (**32**) was 7–19-fold less cytotoxic than RA-VII (**1**), and RA-XXVI (**33**) was 7–12-fold less cytotoxic than deoxybouvardin (**3**). Comparison of the X-ray crystal structures of RA-VII (**1**), RA-XXV (**32**), and RA-XXVI acetate (**34**) revealed that the backbone structure of **34** bears a good resemblance to that of **1**, whereas the backbone structure of **32** does not. Investigation of their conformational features in solution showed that peptides **32** and **33** preferentially adopt a backbone conformation not seen in known natural peptides of this series, and the population of the active conformer is small. Thus, the *N*-methyl group at Tyr-5 is necessary for this series of peptides to take the active conformation preferentially.

### Allo-RA-V, neo-RA-V, and *O*-seco-RA-V

**Fig. 10:**
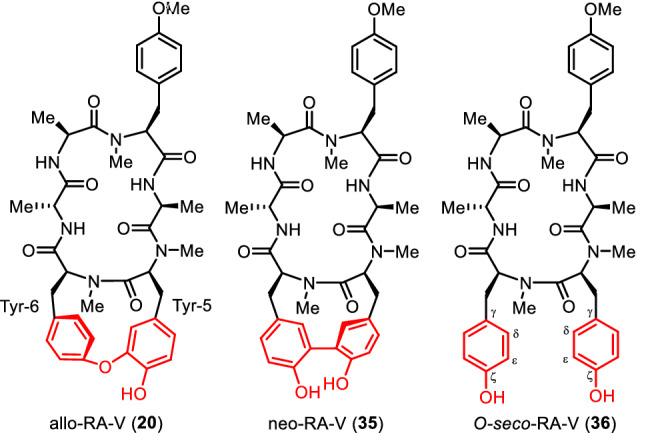
Structures of allo-RA-V, neo-RA-V, and *O*-seco-RA-V

In typical RA-series peptides including RA-VII (**1**) and deoxybouvardin (**3**), an ether linkage is formed between the carbon atom at the ζ position of Tyr-5 and the carbon atom at the ε position of Tyr-6, whereas in allo-RA-V (**20**), the ether linkage is formed between the carbon atom at the ε position of Tyr-5 and the carbon atom at the ζ position of Tyr-6 to form a cycloisodityrosine structure. In neo-RA-V (**35**), those two tyrosines, Tyr-5 and Tyr-6 linked by a C–C bond between their ε positions to form an unusual 12-membered cyclodityrosine structure. *O*-seco-RA-V (**36**) is a monocyclic peptide that has been reported as a synthetically known compound but was isolated from natural sources for the first time [34] (Fig. 10).

**Fig. 11:**
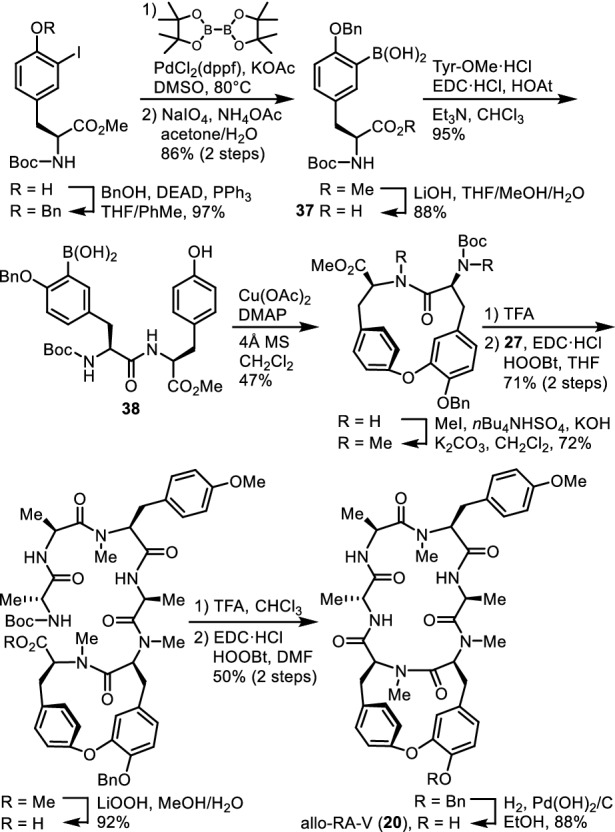
Synthesis of allo-RA-V (**20**)

**Fig. 12:**
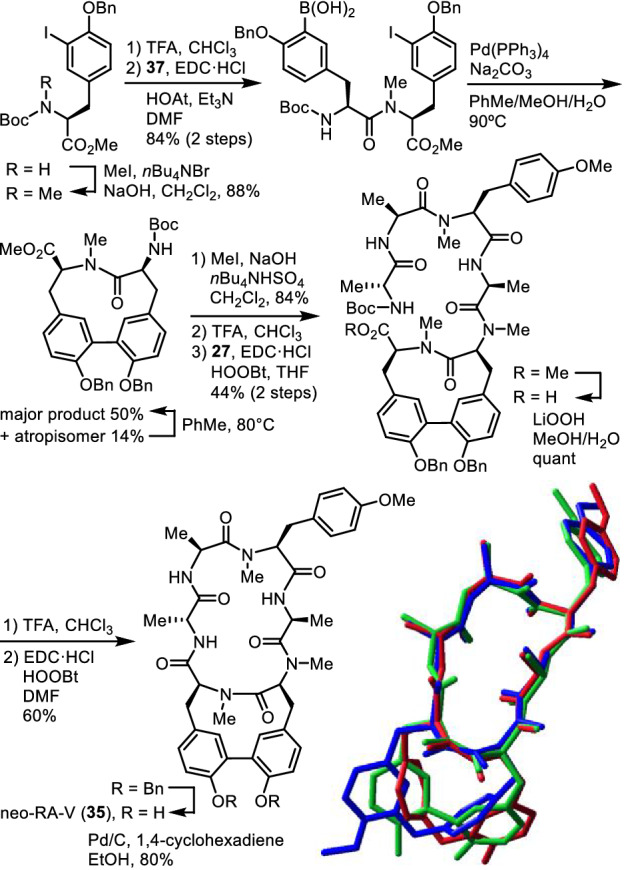
Synthesis of neo-RA-V (**35**) and superposition of the crystal structures of RA-VII (**1**, blue) and neo-RA-V (**35**, green), and the energy-minimized structure of allo-RA-V (**20**, red)

Allo-RA-V (**20**) was synthesized by employing a similar synthetic pathway to that for the synthesis of analogues **23a,b** and RA-XXV (**32**). The cycloisodityrosine unit of allo-RA-V (**20**) was prepared by cyclization of modified tyrosyl-tyrosine **38** in which the boronic acid group existed at the N-terminal tyrosine (Fig. 11). The 12-membered cyclodityrosine unit of neo-RA-V (**35**) was synthesized through an intramolecular Suzuki–Miyaura cross-coupling reaction (Fig. 12). Allo-RA-V (**20**) was 790–1000-fold less cytotoxic than RA-VII (**1**) and 600–830-fold less cytotoxic than deoxybouvardin (**3**) towards HL-60 and HCT-116 cell lines, whereas neo-RA-V (**35**) and *O*-seco-RA-V (**36**) exhibited almost no cytotoxicity towards those cell lines. Comparison of the 3D structures of highly active RA-VII (**1**) with less active allo-RA-V (**20**) and neo-RA-V (**35**) suggests that the orientation of the Tyr-5 and/or Tyr-6 phenyl rings plays a significant role in their biological activity (Fig. 12). The isolation of peptides **20**, **35**, and **36**, along with compound **3**, and the comparison of their structures seem to indicate that peptide **36** may be the common precursor for bicyclic peptides **3**, **20**, and **35** in the plant.

### RA-dimer B

**Fig. 13:**
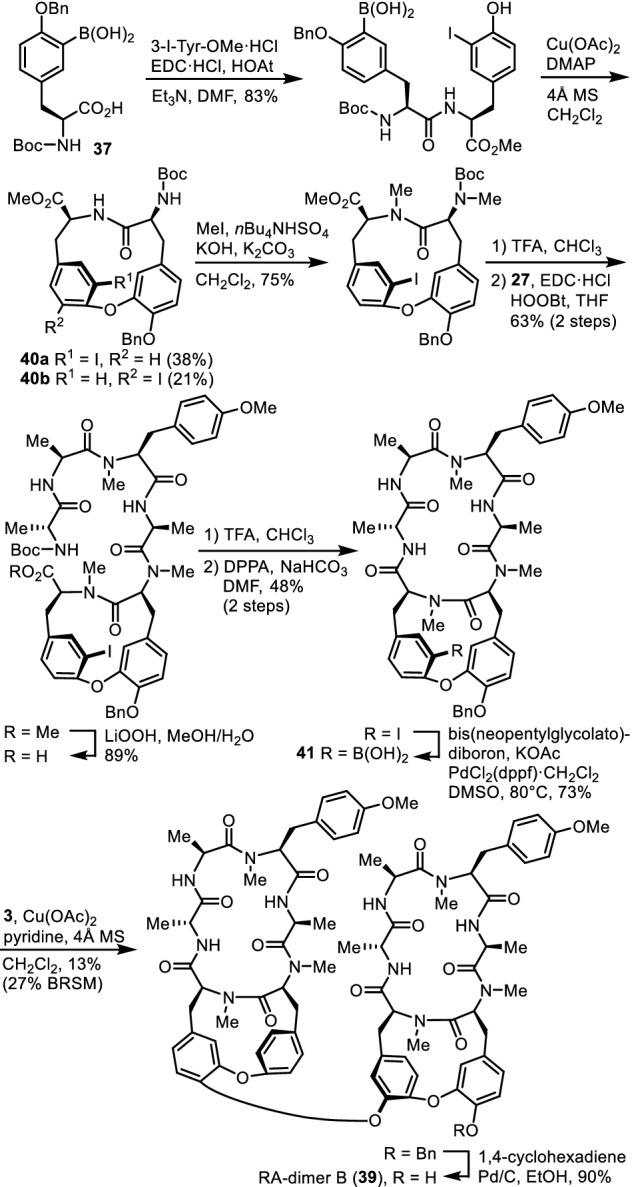
Synthesis of RA-dimer B (**39**)

We have previously isolated RA-dimer A, a dimer of deoxybouvardin (**3**), in which two deoxybouvardin molecules are connected between the phenolic oxygen atom of one deoxybouvardin and the εa-carbon atom of the Tyr-6 residue of the other deoxybouvardin [38]. RA-dimer B (**39**), the second dimeric RA-series peptide, is composed of deoxybouvardin (**3**) and allo-RA-V (**20**), and those two cyclopeptides are connected between the phenolic oxygen atom of deoxybouvardin and the εa-carbon atom of the Tyr-6 residue of allo-RA-V (**20**) (Fig. 13) [39]. The structure of RA-dimer B (**39**) was elucidated on the basis of spectroscopic data, and the synthesis of **39** confirmed the relative stereochemistry and established the absolute configuration of this peptide. Peptide **39** was synthesized by a coupling reaction of deoxybouvardin (**3**) with the boronic acid derivative of allo-RA-V, **41**, and subsequent deprotection. Installation of the boronic acid functionality at the proper position of the allo-RA-V molecule was achieved by substitution of the pre-installed iodine atom by a boronic acid group as in the synthesis of boryl tyrosine **17**. In the synthesis of **41**, the intermediate iodocycloisodityrosine was obtained as a separable mixture of atropisomers **40a** and **40b**, and desired isomer **40a** was then converted into boronic acid intermediate **41**. Peptide **39** was 54-fold and 95-fold less cytotoxic than RA-VII (**1**) towards HL-60 and HCT-116 cell lines, respectively.

## Conclusion

We have developed an efficient method to prepare 14-membered cycloisodityrosines from commercially available l-tyrosine derivatives, and synthesized several RA-VII analogues and new RA-series peptides with proposed structures. Such a totally synthetic approach will allow access to RA-VII analogues not available from natural peptides, which will provide valuable information for the structure–activity relationship studies of RA-VII, being useful for the design of analogues, some of which may express more promising biological properties. The chemical syntheses of natural peptides enabled us to establish the correct structures of them.
